# Aspergilloma in sarcoidosis

**DOI:** 10.4103/0970-2113.56347

**Published:** 2009

**Authors:** Jagruti Hede, Rahul Bahot, J. R. Shah

**Affiliations:** *Department of Chest Diseases, Jaslok Hospital and Research Centre, Mumbai - 400 026, India*

**Keywords:** Aspergilloma, sarcoidosis, tuberculosis

## Abstract

This is a case report of a 48-year-old man, followed up for nearly 30 years who initially developed sarcoidosis at the age of 18 that went into remission after 8 years of treatment. Ten years later, he developed sputum-positive tuberculosis and was cured with anti-tubercular treatment. Following this, there was progression of sarcoidosis to stage IV fibrocystic disease. Ten years later, he had massive hemoptysis during which time, aspergilloma was detected in a sarcoid cystic cavity.

## INTRODUCTION

Aspergilloma is generally seen in tubercular cavities. To the best of our knowledge, aspergilloma in sarcoidosis has not been reported in Indian literature.

We present here the case report of a patient first seen in 1980 and diagnosed with stage II sarcoidosis. He was treated with steroids for 8 years until he was considered clinically cured. He remained in good health for 10 years. In 1998, he developed sputum positive for tuberculosis, which cleared out completely with anti-tubercular treatment, but there was progression of original sarcoidosis to stage IV fibrocystic disease. Six years later, in 2004, he developed massive hemoptysis that led to the detection of aspergilloma in sarcoid cystic cavity.

When the patient first presented to us with stage I disease of bilateral hilar lymphadenopathy, negative tuberculin test and increased serum angiotensin-converting enzyme (ACE) levels, there was no other diagnosis considered. During the last 20 years, this patient who is a medical practitioner has been under careful follow-up and has gone through all classical 4 stages of sarcoidosis as determined by the chest X-ray and computed tomography (CT) scan [Stage I of bilateral hilar lymphadenopathy, stage II of bilateral hilar lymphadenopathy and pulmonary infiltrates, stage III of only pulmonary infiltrates and stage IV of fibrocystic sarcoidosis], making it impossible to entertain any other diagnosis.

The interesting points of this case report are the occurrence of aspergilloma in sarcoid cystic cavity; with concomitant tuberculosis infection and sarcoidosis; the very adverse prognosis of aspergilloma in sarcoid cavity unlike aspergilloma in tubercular cavity; therapeutic problems of management and nearly 30 years of meticulous follow-up.

## CASE REPORT

### 1980-1988

**Figure 1:**
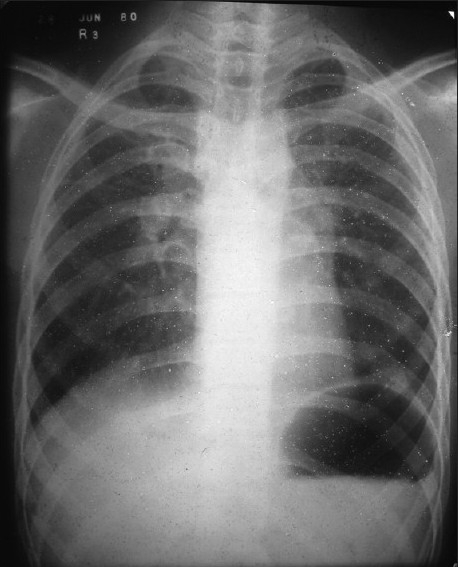
Chest X-ray (1980) showing bilateral hilar and left paratracheal lymphadenopathy with granular infiltrates in both upper lobes

The patient, a 48-year-old practicing homeopath, first came for consultation to the department at the age of 18 years. He was suffering from persistent dry cough for one and half years and progressively worsening breathlessness on exertion for three months. He had no fever, loss of weight or other constitutional symptoms. The routine investigations were normal and the 5 purified protein derivative (PPD) Mantoux test was negative. The lung function tests showed restrictive defect of ventilation. The arterial blood gases showed hypoxemia. The chest X-rays taken during the period showed bilateral hilar and left paratracheal lymphadenopathy and, later, development of fresh scattered granulomatous infiltrate in the both upper zones, with more on the right side [Figure 1].

The patient was diagnosed to be suffering from stage II sarcoidosis on the basis of persistent and progressively increasing severity of cough and breathlessness, chest X-rays showing bilateral hilar and left paratracheal lymphadenopathy, with gradually increasing microgranular infiltrates in both upper zones, high serum ACE levels and negative Mantoux test. He was given tablet prednisolone 30 mg/day, which was tapered off after 10 months and subsequently maintained on 10 mg on alternate days. Follow-up chest X-rays showed total regression of bilateral hilar lymphadenopathy but persistence of scattered granulomatous lesions in both upper zones.

Toward 1988, the chest X-rays had cleared reasonably well with residual minimal micronodular opacities in both upper zones. At this time, prednisolone was discontinued.

### 1988-1998

He remained healthy and in good physical fitness. During these 10 years, he did not take any treatment. The chest X-rays showed no further change.

### 1998-2001

**Figure 2:**
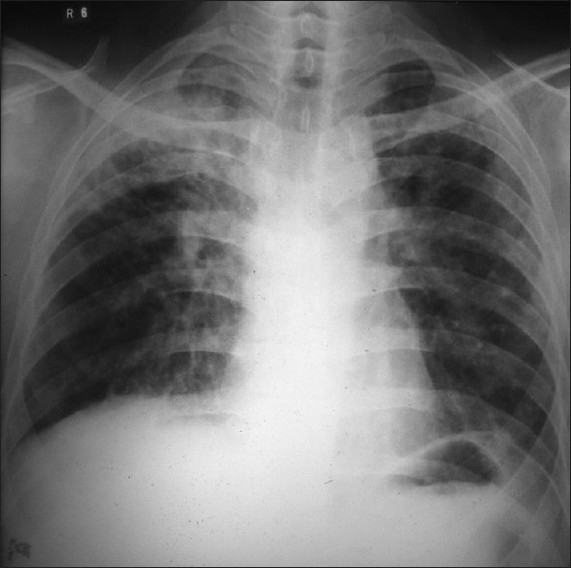
Chest X-ray (1998) shows right tubercular exudative lesions with cavity in right upper lobe

He developed fever, cough with expectoration and loss of weight in the mid 1998. The chest X-ray showed dense exudative lesions occupying almost the entire right upper lobe, with a large cavity. All 3 samples of sputum were positive for AFB (++). He was given a 4-drug anti-tubercular treatment. During the course of the treatment, the chest X-ray showed progressive clearance of dense lesions with closure of the cavity. At the end of 10 months, only residual scarring was seen [Figure 2].

After about 6 months of good health following the completion of anti-tubercular treatment and the closure of the cavity, he developed progressively increasing breathlessness. The lung function tests showed deterioration over earlier results. The arterial blood gases also showed increase in hypoxemia. The chest X-ray showed reduced volume of lungs and bilateral extensive interstitial fibronodular pattern. The chest CT scan showed fibrocystic disease with scattered micronodular pattern. He was restarted on tablet prednisolone 10 mg daily, which provided good relief.

### 2001-2004

The patient's breathlessness gradually increased in severity, restricting his daily activities. He also developed resting cyanosis. The arterial blood gases showed severe hypoxemia (PaO_2_ 49.4 mmHg) with significant desaturation after exercise. The lung function tests showed severe restrictive defect of ventilation, reduced diffusing capacity and with some reversibility in small airways postbronchodilator. The serum ACE level was increased to 60 U/L.

**Figure 3:**
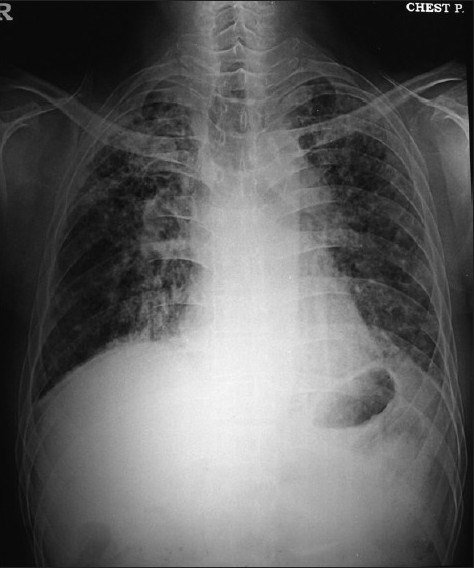
Chest X-ray (2007) shows exudative interstitial nodular and fibrotic pattern

The repeat chest X-ray [Figure 3] and the CT scan of the chest done at this time showed further progression of extensive fibrocystic disease.

### 2004-2008

**Figure 4:**
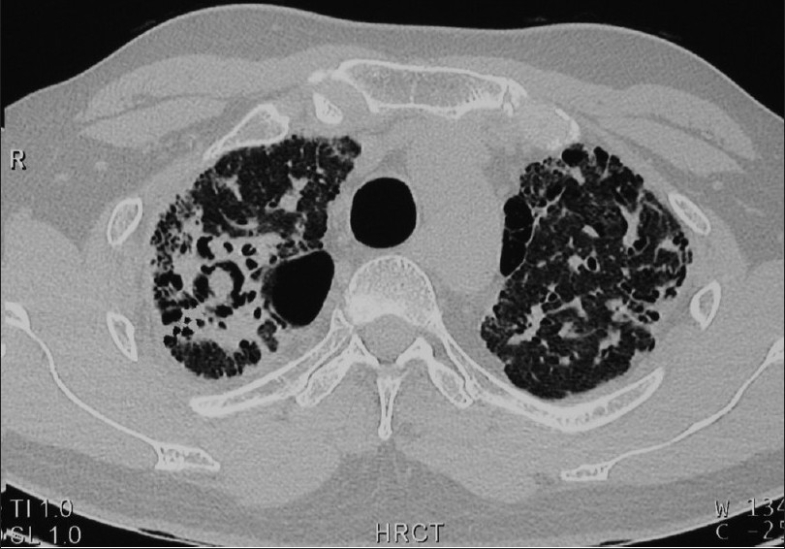
HRCT slide (2007) showing aspergilloma in the right upper lobar cavity and extensive fibrosis in both lung fields

He developed two bouts of hemoptysis in mid 2004 and was admitted into the ICU of another hospital where he was intubated and put on a ventilator. He was discharged on good recovery. His chest X-ray and chest CT scan showed reduced lung volumes, extensive interalveolar septal fibrosis extending to both lung fields with few thin-walled cysts in both lung fields, aspergilloma in the right upper lobe cystic cavity. Retrospectively, the beginning of nidus of aspergilloma was also seen in the cyst on the CT scan done in 1998 [Figure 4].

He was generally breathless, moved about in a wheel chair, required oxygen for nearly 20 hours daily. His tablet prednisolone has increased to 15 mg daily but his general health was gradually going down and he could not move about without oxygen.

### 2008

The patient was last seen this year in our department for the general worsening of his physical condition resulting from lower respiratory tract infection. He had persistent cough with mucopurulent expectoration and showed gross cushingoid features, finger clubbing, cyanosis, with tachypnea 34/min and tachycardia 104/min. His oxygen saturation was 64% on room air and increased to 86% on oxygen 4 L/min. The serum precipitin antibodies to *Aspergillus* were positive (116 U) [Positive: Above 12.0 U].

Daily steroids were replaced by intramuscular administration of Depomedrol 40 mg once a week. In addition, tablet sildenafil, antibiotics and antifungal were also added. He showed considerable and satisfactory improvement on this regime.

## DISCUSSION

In the environment, *Aspergillus* produces small spores, which when inhaled, may result in saprophytic colonization. Aspergilloma (mycetoma) is a mass of fungal mycelia, tissue debris and inflammatory cells. It occurs in pre-existing cavities resulting from tuberculosis, bronchiectasis or sarcoidosis. Aspergilloma is generally detected on routine chest imaging. Typical imaging shows an upper lobe cavity containing irregular outlined mass opacity, which is mobile with change in position.[1]

The diagnosis of aspergilloma is usually made on the basis of clinical and chest radiographic features coupled with serologic evidence of precipitating antibodies to *Aspergillus* spp.[2]

However, a large majority of patients have recurrent mild hemoptysis. Aspergilloma is also known to cause massive hemoptysis by local invasion of bronchial vessel.[3] In a review article on the follow-up of aspergilloma in 14 patients with sarcoidosis and 14 with tuberculosis, authors concluded poor prognosis in both groups.[4]

Although the risk of morbidity and mortality is high, surgical resection of cavity for the prevention of massive hemoptysis is possible in tubercular cavity.[1,5] However, it cannot be considered for sarcoidosis because of poor pulmonary reserve. Close supervision is necessary and in an event of hemoptysis, embolization should be considered.[3]
